# Immunity of Foot-and-Mouth Disease Serotype Asia 1 by Sublingual Vaccination

**DOI:** 10.1371/journal.pone.0063839

**Published:** 2013-05-22

**Authors:** Hao-tai Chen, Yong-sheng Liu

**Affiliations:** State Key Laboratory of Veterinary Etiologic Biology, National Foot-and-Mouth Disease Reference Laboratory of China, Lanzhou Veterinary Research Institute, Chinese Academy of Agricultural Sciences, Lanzhou, Gansu, P. R. China; Public Health Agency of Canada, Canada

## Abstract

Foot-and-mouth disease virus (FMDV) causes vesicular disease of cloven-hoofed animals, with severe agricultural and economic losses. Here we present study using a sublingual (SL) route with the killed serotype Asia 1 FMDV vaccine. Guinea pigs were vaccinated using a commercially available vaccine formulation at the manufacturer’s recommended full, 1/4, and 1/16 antigen doses. Animals were challenged with homologous FMDV Asia1 strain at various times following vaccination. All control guinea pigs exhibited clinical disease, including fever, viremia, and lesions, specifically vesicle formation in feet. Animals vaccinated with the 1/16 and 1/4 doses were protected after challenge at days 7, 28, and 35 post vaccination. These data suggest that effective protection against foot-and-mouth disease can be achieved with 1/16 of the recommended vaccine dose using SL vaccination, indicating that the sublingual route is an attractive alternative for the administration of the FMDV vaccine.

## Introduction

Foot-and-mouth disease virus (FMDV) is a member of the genus *Aphthovirus* of the family *Picornaviridae*, which is divided into seven serotypes with no cross-protection conferred among the serotypes [1]. FMDV serotypes O and A are widely distributed worldwide, but serotypes C has not been observed for years. Interestedly FMDV serotypes SAT 1, SAT 2, SAT 3 are normally restricted to Africa and FMDV serotype Asia 1 to Asia [2], [3]. Due to the aggressive FMD nature, outbreaks usually result in severe economic losses and impact on both national and international trade within the livestock and animal products [4]–[14]. Vaccination of any suspected FMD cases is of utmost urgency to control this veterinary infection given the extreme contagiousness of the causative virus.

In the present study, we evaluated the response to SL inoculation of killed virus vaccine. We tested for protection against live virus challenge at the days 28 and 35 following vaccination as well as induction of rapid protection by challenging at 7 days after vaccination. Results indicated that the use of SL vaccination for rapid protection, such as during outbreaks of FMD in disease free countries and for standard vaccination utility in eradication programs.

## Materials and Methods

### Animals

Guinea pigs (Lanzhou veterinary research institute, China) weighing at 200–300 g were maintained under pathogen-free conditions with free access to pathogen-free food and water. All guinea pig experiments were performed in a bio-safety level 3 animal facilities of State Key Laboratory of Veterinary Etiologic Biology following the protocol approved China Institutional Animal Use and Care Committee. Animal temperatures and serum were taken before any inoculations.

### Vaccine

A commercial vaccine was provided following standard manufacturing protocols, using the inactivated and purified FMDV Asia1/CHA/2005 strain. The FMDV 146 s antigen was formulated with saponin as adjuvant and formulated according to vaccine standards. A mock vaccine containing no antigen in the saponin, was also prepared. The vaccines were administered at the different doses in 0.2 ml per guinea pigs.

### Vaccination

To confirm the FMDV vaccine protects guinea pigs, the standard dose of the killed virus vaccine was adjusted to a 0.2 ml volume for SL delivery. Further we tested full, 1/4 and 1/16 antigen doses. In trial A, 5 guinea pigs were vaccinated with each different formulation and then challenged at 28 days post-vaccination (Table 1).

**Table 1:** Challenge at 28 days post-vaccination.

Vaccine formulation		Groups	Animalnumbers	Clinical scoring				Lameness/Fever
Antigen	Adjuvant			Day 1	Day 4	Day 7	Day 10	
1	1	A1	A1–1	0	0	0	0	−/−
			A1–2	0	0.5	0.5	0.5	−/−
			A1–3	0	0	0	0	−/−
			A1–4	0	0	0	0	−/−
			A1–5	0	0	0	0	−/−
1	1/2	A2	A2–1	0	0	0	0	−/−
			A2–2	0	0	0	0	−/−
			A2–3	0	0	0	0	−/−
			A2–4	0	0	0	0	−/−
			A2–5	0	0	0	0	−/−
1/4	1/2	A3.	A3–1	0	0	0	0	−/−
			A3–2	0	0.5	0.5	0.5	−/−
			A3–3	0	0	0	0	−/−
			A3–4	0	0	0	0	−/−
			A3–5	0	0	0	0	−/−
PBS	PBS	A4	A4–1	0	3.5	3.5	3.5	+/+
			A4–2	0	3.5	3.5	3.5	+/+
			A4–3	0	3.5	3.5	3.5	+/+

In trial B, we tested delivery full, 1/4 and 1/16 doses of killed virus antigen by SL delivery. 5 animals were tested at each above dose, and challenged at 7 days post-vaccination (Table 2).

**Table 2:** Challenge at 7 days post-vaccination.

Vaccine formulation		Groups	Animalnumbers	Clinical scoring				Lameness/Fever
Antigen	Adjuvant			Day 1	Day 4	Day 7	Day 10	
1	1/2	B1	B1–1	0	0	0	0	−/−
			B1–2	0	0.5	0.5	0.5	−/−
			B1–3	0	0	0	0	−/−
			B1–4	0	0	0	0	−/−
			B1–5	0	0	0	0	−/−
1/4	1/2	B2	B2–1	0	0	0	0	−/−
			B2–2	0	0	0	0	−/−
			B2–3	0	0	0	0	−/−
			B2–4	0	0	0	0	−/−
			B2–5	0	0	0	0	−/−
1/16	1/2	B3	B3–1	0	0	0	0	−/−
			B3–2	0	0	0	0	−/−
			B3–3	0	0.5	0.5	0.5	−/−
			B3–4	0	0	0	0	−/−
			B3–5	0	0	0	0	−/−
PBS	PBS	B4	B4–1	0	3.5	3.5	3.5	+/+
			B4–2	0	3.5	3.5	3.5	+/+
			B4–3	0	3.5	3.5	3.5	+/+

In trial C, 5 guinea pigs were vaccinated as above test at 35 days. 5 guinea pigs were vaccinated with either 1/4, 1/16, or mock vaccine (Table 3). Each experiment included three naïve animals used as controls.

**Table 3:** Challenge at 35 days post vaccination.

Vaccine formulation		Groups	Animalnumbers	Clinical scoring				Lameness/Fever
Antigen	Adjuvant			Day 1	Day 4	Day 7	Day 10	
1	1	C1	C1–1	0	0	0	0	−/−
			C1–2	0	0	0	0	−/−
			C1–3	0	0	0	0	−/−
			C1–4	0	0	0	0	−/−
			C1–5	0	0	0	0	−/−
1	1/2	C2	C2–1	0	0	0	0	−/−
			C2–2	0	0	0	0	−/−
			C2–3	0	0	0	0	−/−
			C2–4	0	0	0	0	−/−
			C2–5	0	0	0	0	−/−
1/4	1/2	C3	C3–1	0	0	0	0	−/−
			C3–2	0.5	0.5	0.5	0.5	−/−
			C3–3	0	0	0	0	−/−
			C3–4	0	0	0	0	−/−
			C3–5	0	0	0	0	−/−
Mock	Mock	C4	C4–1	0	3.5	3.5	3.5	+/+
			C4–2	0	3.5	3.5	3.5	+/+
			C4–3	0	3.5	3.5	3.5	+/+

### Challenge

The challenge virus was isolated and harvested from the animal infected with FMDV Asia1/CHA/2005 strain. The challenge virus was titrated to determine 50% guinea pig infectious dose (GPID50). Virus aliquots were maintained and stored at −70°C until use.

Guinea pigs were challenged with 100 GPID50. Animals that showed the vesicles only at the original injected site (clinical score 0.5) were judged to be protected, and those that showed any FMD clinical signs in the other three feet were judged to be unprotected.

### Clinical Measurement

Guinea pigs were monitored for clinical signs of FMD during the vaccination and challenge periods. Temperatures were recorded daily for each experiment. Animals were examined kindly for clinical lesions at days 0, 4, 7, and 10 post-challenge. A clinical score was determined based on the number of the three non-injected feet. The maximum clinical score is 3.5 including the score 0.5 in the injected foot.

### Serum Neutralizing Antibodies

Serum samples were tested for the presence of neutralizing antibodies against FMDV by a standard protocol. Serum samples were heat inactivated at 56°C for 30 min. Serial dilutions were incubated with 100 TCID50 of FMDV for 1 h at 37°C. These samples were then transferred to BHK-21 cells and incubated at 37°C for 72 h. Cytopathic effect (CPE) was microscopically determined that the endpoint titers were the reciprocal of the last serum dilution to neutralize virus in 50% of the wells [15].

### Virus Titration

Virus titers in serum were established by determining TCID50. Briefly, 10-fold serial dilutions of serum were added to BHK-21 cells in a 96-well microtiter plate, four replicates per dilution. Tissue culture plates were incubated at 37°C for 72 h and monitored for CPE in order to calculate TCID50.

## Results

### Clinical Assessments

**Figure 1:**
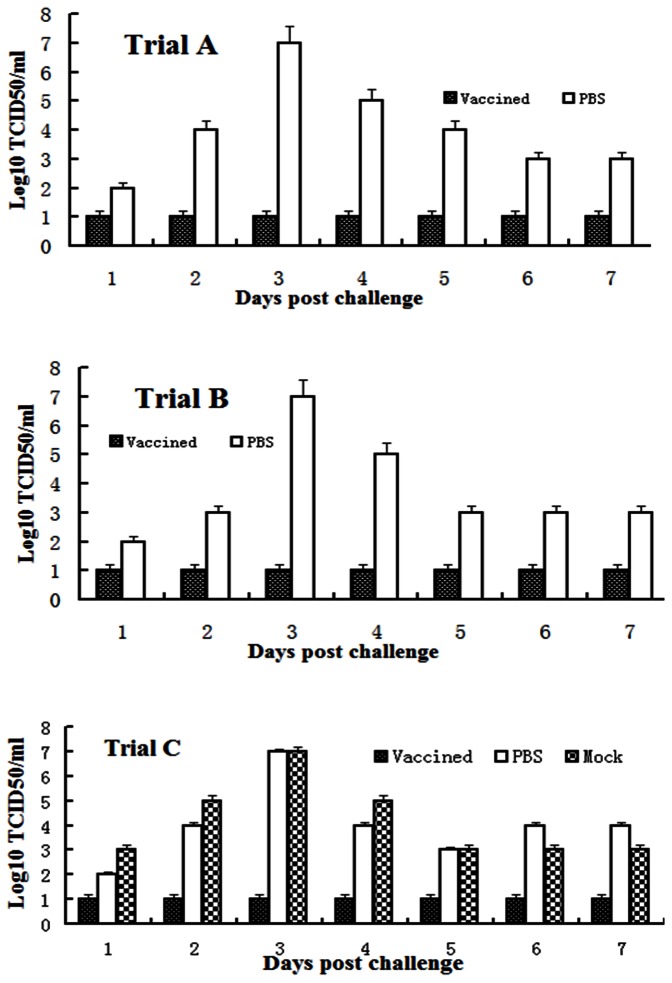
Virus isolation of the guinea pig serum samples from one to seven days post challenge is shown in Trial A (Fig.1A), Trial B (Fig. 1B) and Trial C (Fig. 1C). Control animals showed viremia peaking at day 3 and were undetected at day 5 post-challenge. Virus titers were established by determining TCID50. Averages standard deviation are shown.

Immune responses elicited by the FMDV vaccine, were evaluated at several dosages. In trial A, all guinea pigs were housed in the same settings and challenged at the same time, including 3 naïve controls. All vaccinated animals were protected from the FMDV infection after challenge at 28 days post-vaccination (Table 1). There was no clinical sign (Table 1) and no viremia (Fig. 1A) exhibited for all the vaccinated animals. Naïve animals showed clinical signs at 2–3 days post-challenge and viremia that peaked at day 3 (Fig. 1A). Viremia and clinical signs resolved at 5 days post-challenge and vesicular lesions started to heal at day 10. Additionally SL delivery of FMDV vaccine with saponin adjuvant showed very mild adverse site reactions (data not shown), and this site reaction was similar regardless of the vaccination dose.

The trial B was performed to determine if FMDV protection can be induced as rapidly as the same vaccine. 5 guinea pigs were vaccinated at 7 days prior to challenge. All animals were housed in the above settings, and challenged the same time, including three naïve controls. All of the animals vaccinated with the 1/4 antigen dose were completely protected from clinical disease at days 4, 7 and 10 post- challenge (Table 2).

In the group of animals vaccinated with 1/16 antigen load, all guinea pigs showed no clinical signs. No vaccinated animals had fever or detectable viremia during the trial course. As expected, naïve animals had vesicle formation on all 4 feet at day 3 post-challenge. Fever and lameness were detected at 3 days post challenge in all naïve animals (Table 2),which was able to detect viremia, again peaking at day 3 and resolving at day 5 (Fig. 1B).

Guinea pigs vaccinated at 35 days prior to challenge with 1/16, and1/4 dose showed no the clinical signs of disease. Animals vaccinated at 7 days prior to challenge with 1/4 dose also showed no signs of the clinical disease (Table 3).

All antigen-vaccinated animals were free of clinical signs regardless of antigen dose or challenge time. In trials A and B, no virus was detected in the serum on any day post-challenge with analysis daily through 7 days. Mock groups and naïve animals challenged at either time point were able to observe the vesicle formation on all 4 feet at day 2 post-challenge. All control and mock animals also showed viremia that peaked at day 3 and resolved at day 5 post-challenge (Fig. 1C).

### Neutralizing Antibody Responses

**Figure 2:**
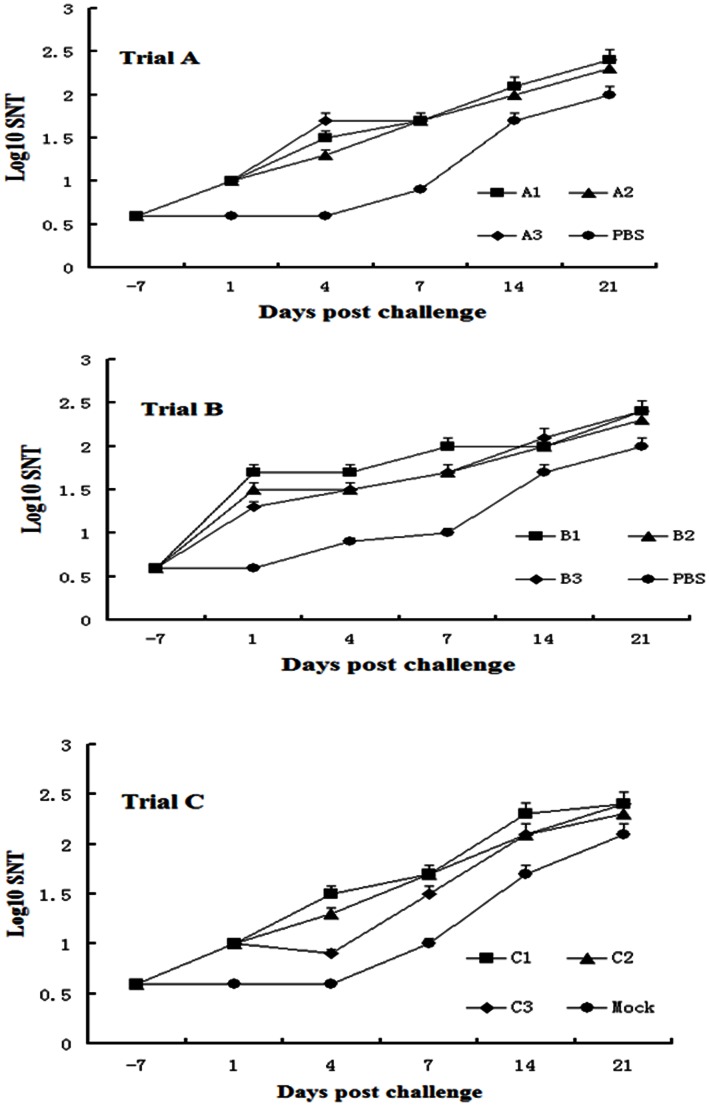
Mean serum neutralizing antibody titers (SNT) are shown in guinea pigs following SL vaccination and challenge with FMDV. Guinea pigs were vaccinated at days 28 (Trial A), 7 (Trial B) and 35 (Trial C) prior to challenge.

All guinea pigs vaccinated with killed virus antigen showed measurable levels of anti-FMDV antibody detected at 7 days following vaccination. Titers of neutralizing antibody in serum were predictive of protection against FMDV (Fig. 2). As has been previously reported, there was increase in neutralizing antibody titer following challenge in all groups, including all control animals.

## Discussion

Developing strategies to control FMD outbreaks is critical to reduce slaughter of livestock and economic losses to farmers. Additionally, the FMDV spread in naïve herds is remarkably rapid and a challenge to control [16], [17]. In present study we have investigated the performance of the killed FMDV vaccine using SL delivery system. The vaccine with the adjuvant saponin was shown to be compatible with this delivery system, as animals show minimal adverse reaction. One important factor in the decision of whether or not to vaccinate in response to an outbreak of FMDV is the daunting logistics of deploying vaccine. Besides matching the vaccine strain to the cycling strain, recruiting professionals to administer vaccine and developing appropriate detection systems to track vaccinated animals is critical. The efficacy of a rapidly applied SL delivery system such as the one tested here will enhance vaccination for FMDV.

Remarkably, the data reported here show that the vaccine SL delivered has elicited immunity response similar to the standard needle delivery of vaccine [4]. In all trials we report here, the vaccine protected against disease. The concentration of viral antigen can be reduced to 1/16 of the standard dose and still provide protection. Concentrations of anti-FMDV antibody that can neutralize virus in vitro were significant at 7 days following vaccination. This result was observed regardless of the viral antigen dose used in the vaccine.

Further, efficacy was not dependent on concentration of the adjuvant saponin. The mock vaccine (adjuvant alone) conferred no protective effects, as mock vaccinated animals were identical in disease assessment to the naïve controls. Fever, lameness and viremia were only detected in the mock and naïve control animals following live virus challenge.

In the past history, formally FMD free countries have seen outbreaks that have raised awareness of the susceptibility of livestock and how rapidly the disease can spread [17], [18]. In 1997, Taiwan virus spread too quickly for vaccination to even be considered, which had a specific tropism for swine [19], no infectivity in cattle [20]. In 2000, there were outbreaks in both South Korea and Japan for the first time in years [21], [22]. Additionally, slaughter was used as the method to eliminate the FMDV infection and regain FMD-free status [21], [23]. In 2001, the United Kingdom suffered a large-scale FMDV outbreak [24], which was a source of disease in Greece, Italy, Ireland and France. All countries slaughtered infected animals and all susceptible animals contacted with the virus. This process was very difficult as millions of animals were slaughtered and quarantine zones had a severe effect on economic activity far beyond the livestock industry. The decision to slaughter infected and exposed livestock was the policy of most of the governments involved for economic reasons. The OIE rules in place called for a 3 months period of no new disease observed before export of animal products was allowed after quarantine and slaughter. If animals were vaccinated, that period was longer, 6 months. The UK outbreak led to a modification of OIE recommendations making the export waiting period 3 months after the last known case of FMDV even if animals were vaccinated to control disease. This change still requires the eventual slaughter of all vaccinated animals. New parameters are being discussed after the FMDV outbreaks in Japan and South Korea. In both cases, vaccine was deployed to help control disease spread. If data from experimental research and FMD outbreaks can confirm newly available tests accurately distinguish between the infected and vaccinated animals, there is support for vaccination strategies to allow vaccinated animals inhibited FMDV infection [25], [26].

Data from this study gives the responsible officials more support for using vaccination to control disease outbreaks. The SL vaccination described here will allow for rapid and safe FMDV vaccination in guinea pigs. In addition, vaccine resources can be expanded as the effective dose for SL vaccination can be lowered compared doses required for FMDV application. More studies with much larger numbers are required to confirm these results and furthermore extended such investigate to large animal host, but the data presented here provide a clear indication of the potential advantage of the SL vaccination.
